# Challenges and advances in two-dimensional photoacoustic computed tomography: a review

**DOI:** 10.1117/1.JBO.29.7.070901

**Published:** 2024-07-12

**Authors:** Shunyao Zhang, Jingyi Miao, Lei S. Li

**Affiliations:** Rice University, Department of Electrical and Computer Engineering, Houston, Texas, United States

**Keywords:** photoacoustic computed tomography, image reconstruction, limited view, anisotropic resolution, acoustic heterogeneity, fluence correction, deep learning

## Abstract

**Significance:**

Photoacoustic computed tomography (PACT), a hybrid imaging modality combining optical excitation with acoustic detection, has rapidly emerged as a prominent biomedical imaging technique.

**Aim:**

We review the challenges and advances of PACT, including (1) limited view, (2) anisotropy resolution, (3) spatial aliasing, (4) acoustic heterogeneity (speed of sound mismatch), and (5) fluence correction of spectral unmixing.

**Approach:**

We performed a comprehensive literature review to summarize the key challenges in PACT toward practical applications and discuss various solutions.

**Results:**

There is a wide range of contributions from both industry and academic spaces. Various approaches, including emerging deep learning methods, are proposed to improve the performance of PACT further.

**Conclusions:**

We outline contemporary technologies aimed at tackling the challenges in PACT applications.

## 1 Introduction

**Fig. 1:**
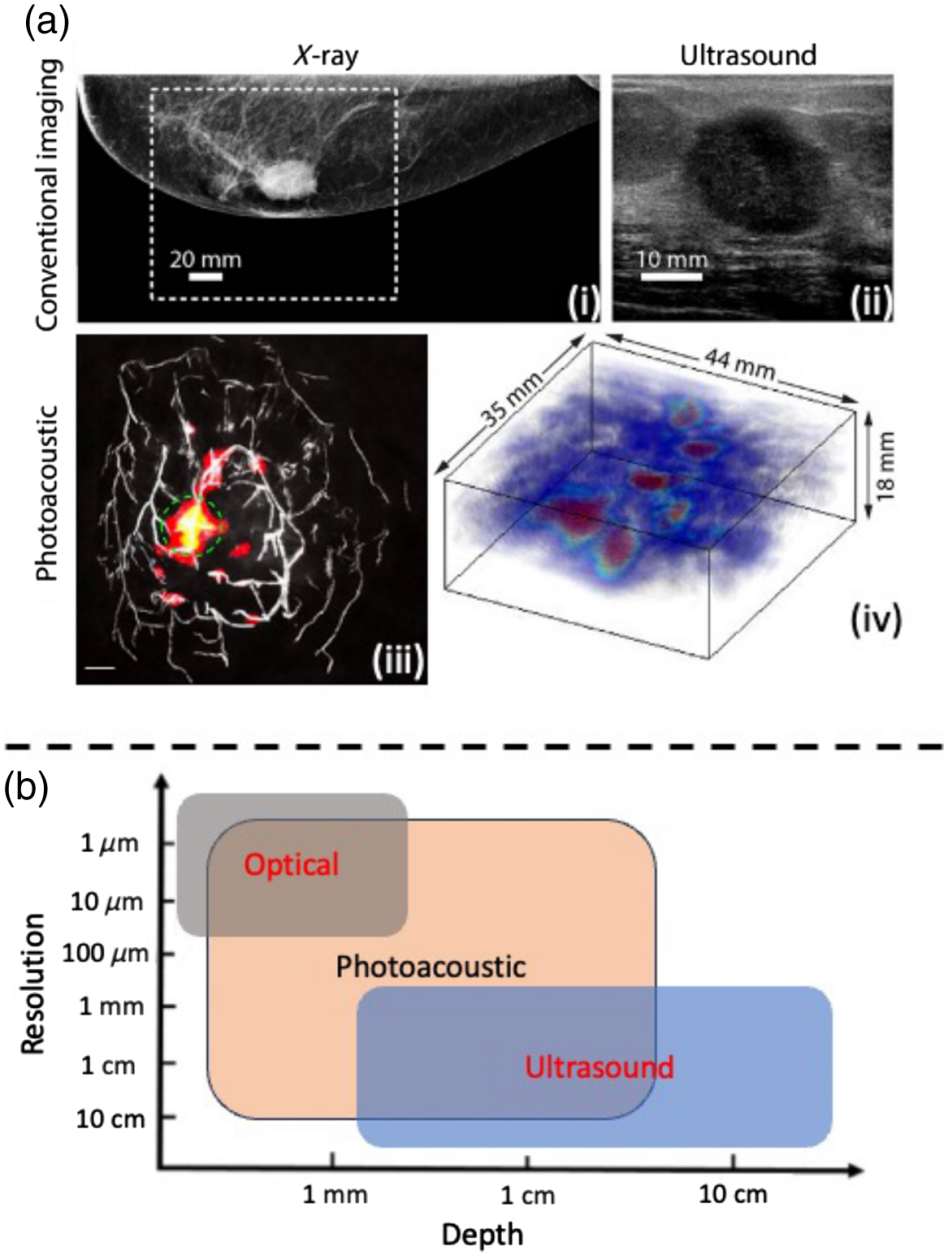
Comparison between X-ray, US, and PA imaging modalities. Reprinted with permission from Refs. 5–7. (a)(i) The X-ray image of the left breast displays a suspicious mass, with the white box indicating the field of view for the PA image. (a)(ii) The US image of the palpable mass confirms a highly suspicious mass. (a)(iii) The MAP of the PA volume depicts vessel density maps with tumors identified by a green circle. (a)(iv) A 3D volume rendering of the PA image exhibits a distinctive ring-like appearance. (b) Evaluation of resolution and depth characteristics across various imaging modalities, including US, optical, and PA imaging.

Biomedical imaging plays a pivotal role in the diagnosis and management of various diseases, offering invaluable insights into the human body’s anatomy and intricate physiological processes.^1^^–^^4^ Traditional imaging modalities, such as X-ray [Fig. 1(a)(i)] and ultrasound (US) [Fig. 1(a)(ii)], have long been the cornerstones of medical diagnostics, each endowed with unique strengths and limitations.^5^^,^^8^^,^^9^ Photoacoustic tomography (PAT)^10^^–^^14^ is a medical imaging technique that employs both optical and acoustic energy, as shown in Fig. 1(a)(iii–iv).^6^ PAT, based on photoacoustic (PA) effect [Fig. 1(b)], transforms absorbed light energy into sound waves.^15^ As shown in Fig. 1(a), PAT, providing high-resolution imaging of breast cancer,^7^^,^^16^^,^^17^ has recently been approved by the Food and Drug Administration as a complementary tool to X-ray mammography and US for breast cancer diagnosis and screening.^18^

**Fig. 2:**
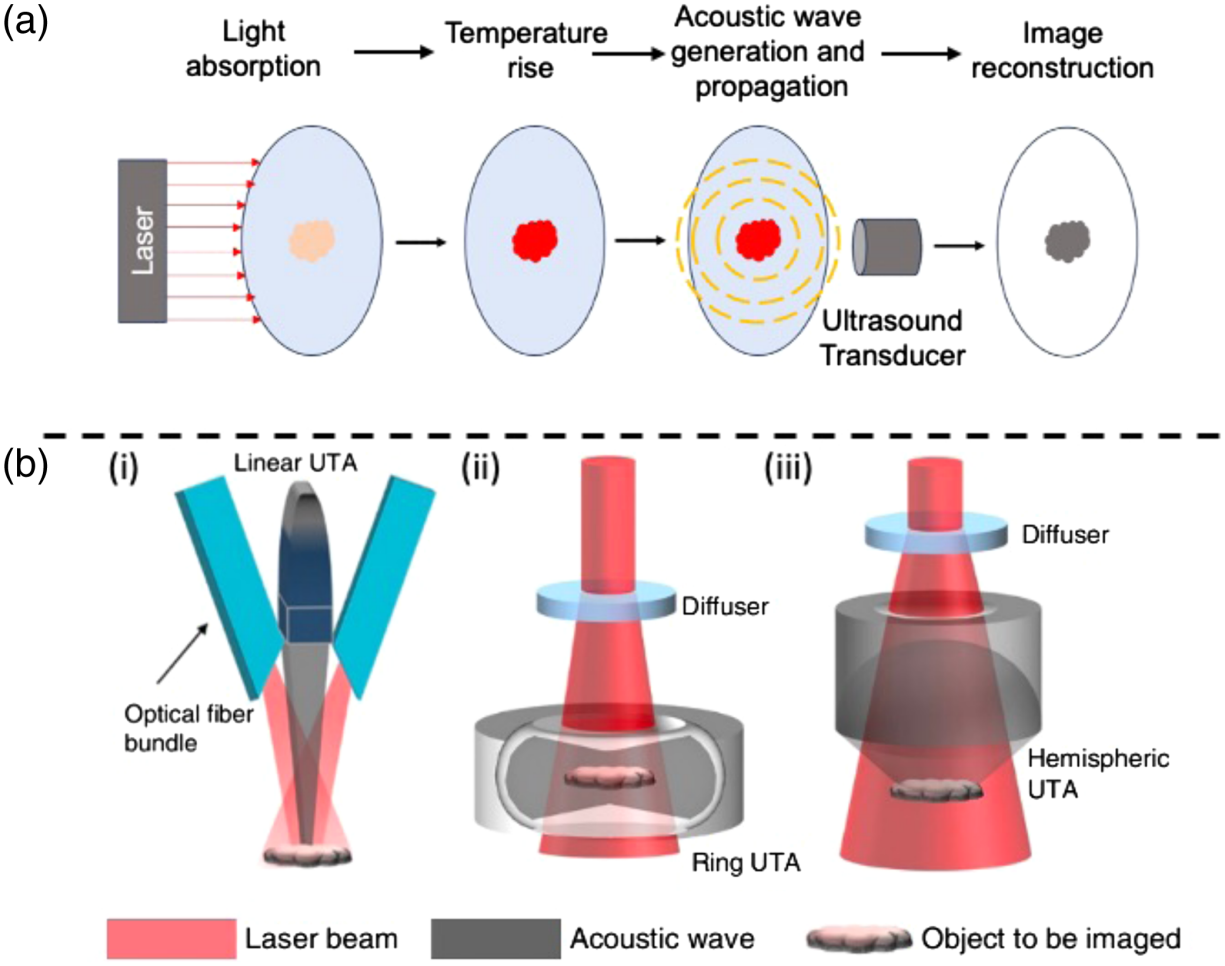
Principle and applications of PACT. Reprinted with permission from Ref. 19. (a) Imaging principle of PACT. (b)(i) PACT system with a linear UTA. (b)(ii) PACT system with a ring-shaped UTA. (b)(iii) PACT system with a hemisphere-shaped UTA.

Figure 2(a) illustrates the principle of PAT. Upon pulsed laser light excitation, temperature arises from the absorption of laser light by tissues, followed by thermal expansion and then the generation of acoustic waves, called PA waves. Ultrasonic transducer array (UTA) detects these waves for image reconstruction (IR).^19^ PA computed tomography (PACT),^20^^–^^25^ a major incarnation of PAT, has enjoyed remarkable progress and widespread adoption in medical imaging in the past 10 years.^7^^,^^26^^–^^32^ PACT utilizes the PA effect, enabling the detection of ultrasonic waves generated by both ballistic and scattered photons excited by a light source. As a result, PACT can penetrate much deeper into tissues compared with traditional optical microscopy, which primarily relies on ballistic photons.^33^^,^^34^ In addition, acoustic waves experience significantly less scattering within soft tissues, leading to PACT offering substantially superior spatial resolution when compared with pure optical imaging methods in deep tissue.^35^ Moreover, thanks to the light–matter interactions, PACT utilizes various molecular contrasts,^36^^–^^44^ including endogenous contrasts, such as hemoglobin, melanin, deoxyribonucleic acid/ribonucleic acid, water, protein, and lipid,^27^^,^^37^^,^^45^^–^^52^ and exogenous contrast agents, such as fluorescent proteins, organic dyes, and nanoparticles.^36^^,^^38^^,^^40^^,^^53^^–^^56^ Understanding the fundamental principles and applications of PACT is crucial for unlocking its full potential, which paves the way for exploring diverse PACT acoustic detection geometries that play a pivotal role in acquiring high-quality images.

PACT employs diverse acoustic detection geometries, including linear, ring-shaped, and hemisphere-shaped arrays [Fig. 2(b)(i–iii)].^19^ While curved UTAs such as ring-shaped and hemispherical arrays can yield high-quality PACT images, they typically require customization and come at a significant expense. In addition, these arrays necessitate accessibility from multiple sides of the target.^57^^,^^58^ On the contrary, linear UTAs can produce images from a single side of the samples, and they are easily accessible at a lower cost, offering the convenience of a handheld approach.^58^^,^^59^ In conclusion, the choice of acoustic detection geometry in PACT depends on the specific application and resource availability.

In this paper, we mainly discuss acoustical inverse problems and an additional optical inverse problem–fluence correction. The acoustical inverse problem involves reconstructing the distribution of initial pressure within the tissue based on the detected acoustic signals, while the optical inverse problem relates to the reconstruction of the optical properties within samples based on measurements of PA signals.

For acoustic inverse problems, practical reconstruction algorithms have been developed. One widely employed approach is the universal back-projection (UBP) algorithm,^60^^–^^62^ where the solid-angle weighting factor is introduced to the back-projection algorithm to compensate for the variations of detection views.^60^ Another alternative algorithm based on the wave physics principle is time reversal (TR).^63^ In TR, the recorded PA signals are mathematically time-reversed and re-emitted into the tissue. As these waves travel back through the tissue, they naturally converge to the location of the original PA source. By detecting and recording the converging waves, an image with optimized spatial resolution and enhanced signal-to-noise ratio (SNR) is generated.^64^^,^^65^ Model-based reconstruction methods have also been developed.^66^^–^^68^ This process involves optimization algorithms that iteratively refine the image by minimizing the least square errors between the measurements and predicted signals according to the exact PA propagation model.^69^^–^^71^

**Fig. 3:**
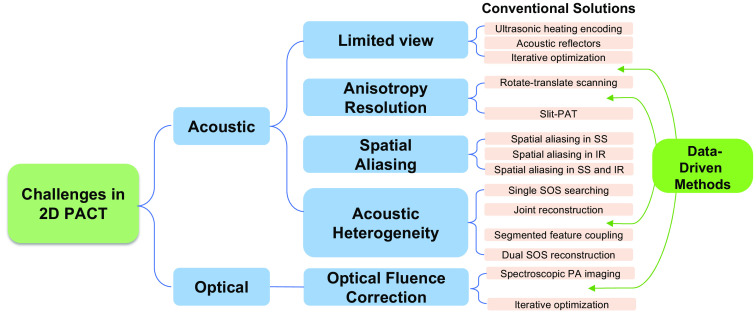
Diagram showing the challenges in 2D PACT and current methods dealing with those challenges. The structure of this review follows this diagram. (SS, spatial sampling; IR, image reconstruction; SOS, speed of sound).

In this review paper, our primary objective is to conduct a thorough examination of some specific challenges inherent to the application of two-dimensional (2D) PACT, as shown in Fig. 3. These challenges include limited view, anisotropy spatial resolution, and acoustic heterogeneity (especially sound speed mismatch) for IR and spectral unmixing with unknown fluence. Through a review of the existing literature, we seek to pinpoint specific hurdles that may impede the full realization of PACT in medical diagnostics. Furthermore, we will identify and dissect research papers and studies that have pioneered innovative solutions to address these challenges. By summarizing and categorizing these solutions, we intend to provide a comprehensive resource for researchers, clinicians, and practitioners eager to harness the capabilities of PACT while effectively mitigating its inherent limitations.

## 2 Hardware/Geometry-Induced Issues

PACT images are reconstructed from the signals recorded by all the elements of the UTA. Thus, different UTA geometries and detector designs of the transducer itself induce issues to the PACT, e.g., limited view, anisotropy resolution, and spatial aliasing.

### 2.1 Limited View and Solutions

**Fig. 4:**
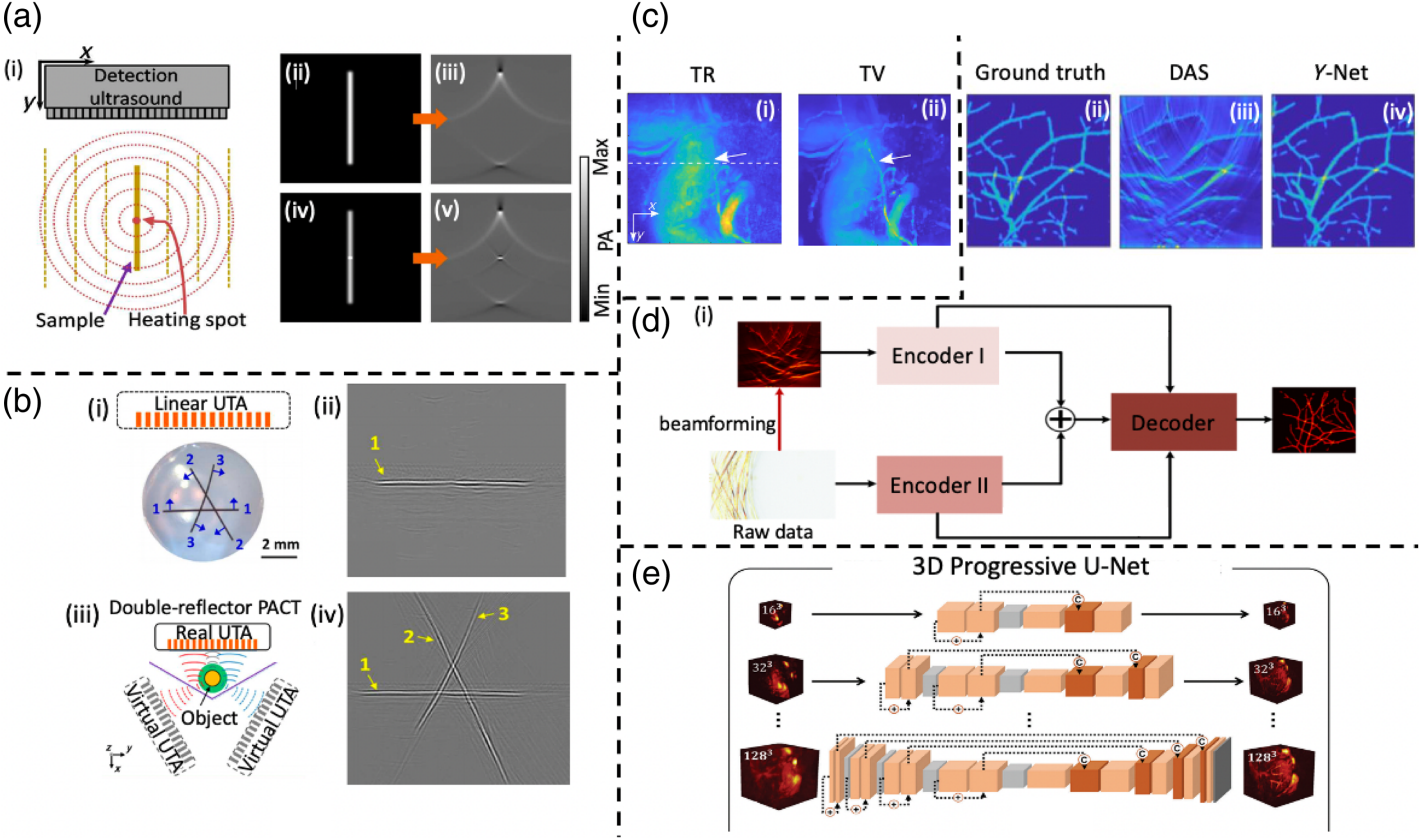
Limited view of challenges and solutions. Reprinted with permission from Refs. 73 and 75–78. (a)(i) Enhanced initial pressure rise at the heating site. (a)(ii) Consistent initial pressure rise across the line phantom. (a)(iii) Reconstructed PA image of line phantom from both ends only. (a)(iv) Ultrasonic heating boosts initial pressure rises at the heated location (center of line phantom). (a)(v) Reconstructed PA image of line phantom from both ends and the center as well. (b)(i) Imaging of a hair phantom with three straight human hairs (labeled as “1–3”). (b)(ii) PA image acquired by conventional PACT. (b)(iii) Two acoustic reflectors are positioned at a relative angle of 120 deg. (b)(iv) PA image acquired by employing double 120-deg acoustic reflector PACT. (c)(i) Human finger joint image reconstructed by non-iterative method—TR. (c)(ii) Human finger joint image reconstructed by iterative method—TV. (d)(i) The global architecture of Y-Net. (d)(ii) Ground truth of initial pressure. (d)(iii) DAS beamformed image. (d)(iv) Reconstructed image from Y-Net. (e) 3D progressive U-Net architecture.

Due to their low cost, hand-held convenience, wide selection of bandwidths, and US imaging capability, linear UTAs have been widely used in PACT to provide real-time cross-sectional images.^58^ However, linear-array-based/planar-array-based systems suffer from the limitation of their viewing angles, resulting in missing features, called the limited view problem.^72^^–^^74^ Linear array detectors exhibit high sensitivity to PA waves propagating perpendicular to the array’s surface. As illustrated in Fig. 4a(i), a linear ultrasonic array is strategically placed orthogonally to a line-shaped numerical phantom. In Fig. 4(a)(ii), the initial pressure rise is visualized. The linear array received PA signals exclusively from the two extremities, displaying the limited view issue. A simple and direct approach to address this issue is to enlarge the detection viewing angles by rotating either the linear array or the object^59^^,^^79^ but sacrificing the imaging temporal resolution. In this section, we review other solutions, including ultrasonic heating encoding, deployment of acoustic reflectors, and advanced deep learning approach.

#### 2.1.1 Ultrasonic heating encoding

The PA amplitude is linearly proportional to the Grueneisen parameter, which is temperature-dependent in various biological tissues; thus, the PA generation can be encoded via temperature encoding. The heat generated by a focused UTA causes a local temperature rise, as depicted in Fig. 4(a)(i), and the Grueneisen parameter at the heated spot is also increased. Then, upon laser light excitation,^80^^–^^82^ the amplitude of the PA signal originating from the heated voxel is higher than the neighboring voxels and remains unchanged, as evidenced in Fig. 4(a)(v). This selective PA signal amplification creates a point PA source, leading to the propagation of PA waves with increased amplitude in all directions. Consequently, these amplitude-enhanced PA waves can be detected by the linear array, addressing the limited view issues. Given the ability to focus ultrasonic heating at considerable depths, this approach holds potential for deep tissue imaging.^75^^,^^83^ Although full-view PACT is demonstrated using ultrasonic heating encoding, there are still some concerns of tissue damage from heating and heat dissipation to surrounding tissues, in turn lowering encoding efficiency.^75^

#### 2.1.2 Acoustic reflectors

To address the limited view issue, employing acoustic reflectors to enlarge the detection view has also been proposed, in turn augmenting the detection coverage angles and recovering the missing features.^72^^,^^76^^,^^84^ Huang et al.^72^ employed a 45-deg acoustic reflector, which acts as a virtual array perpendicular to a physical array. Ellwood et al.^84^ and Li et al.^76^ independently presented an alternative configuration in which two acoustic reflectors were used to increase the effective detection aperture. Experiments^76^ showed that a hair phantom containing three straight human hairs, denoted as “1 to 3,” is subjected to imaging using a linear array detector [Fig. 4(b)(i)]. In Fig. 4(b)(ii), the reconstructed image from conventional linear-array PACT only displayed the horizontal hair “1,” while hairs “2” and “3” were mis-detected due to the limited view. Figure 4(b)(iii) illustrates the configuration of the acoustic reflectors arranged at an enclosed angle of 120 deg. When combined with the reflectors, the detection angle coverage was significantly enhanced, as shown in Fig. 4(b)(iv), and all three hairs were distinctly recovered. One drawback of the acoustic reflector approach is that it constrains imaging space and loses the handheld imaging convenience, making it less suitable for applications requiring larger imaging volumes, such as whole-body imaging of rodents and human imaging.

#### 2.1.3 Iterative optimization

Model-based iterative IR methods have recently been explored to address limited view issues with planar detection geometry in PACT as well.^73^ The image reconstructed from the TR method exhibits that the small vessel indicated by the white arrow is poorly visualized, which is caused by limited view problems, as depicted in Fig. 4(c)(i). Least squares minimization-based iterative approaches were evaluated using the same *in vivo* data. It shows that the small vessel can be clearly visualized in the reconstructed image by the total variation (TV) regularization method, where the missing features are well recovered, as shown in Fig. 4(c)(ii).

#### 2.1.4 Deep learning

Deep learning (DL) methods have been increasingly popular in various PA applications, including exploring the limited view issue.^77^^,^^78^^,^^85^^–^^89^ The delay-and-sum (DAS) beamformed image, as shown in Fig. 4(d)(iii), acquired from a linear array-based PACT, missed a lot of features (especially the vertical vessels) due to the limited view. To tackle this problem, a supervised learning model based on Y-Net architecture [Fig. 4(d)(i)] has been developed. The proposed Y-Net inputs the raw PA signals to encoder II and processes the raw data to obtain an imperfect beamformed image as the input of encoder I, where encoders I and II encode the texture and physical features, respectively, to realize hybrid reconstruction.^89^ Finally, the reconstructed vessel structure [Fig. 4(d)(iv)] resides near ground truth [Fig. 4(d)(ii)], and the shape is well-preserved. The above results demonstrate obvious improvements over DAS reconstruction; however, this work has not been generalized to *in vivo* applications.^77^ Besides, Choi et al.^78^ developed a three-dimensional (3D) progressive U-Net [Fig. 4(e)] to address limited view issues and produced volumetric PACT images by improving the solid angle range by 3.77 times, and then, missing features were well recovered. The performance was successfully demonstrated *in vivo*.^78^ DL methods show promise in enhancing IR accuracy for limited view PACT, but the effectiveness of DL reconstruction is highly sensitive to the quality of training data.^86^^–^^88^^,^^90^

### 2.2 Anisotropy Resolution Solutions

**Fig. 5:**
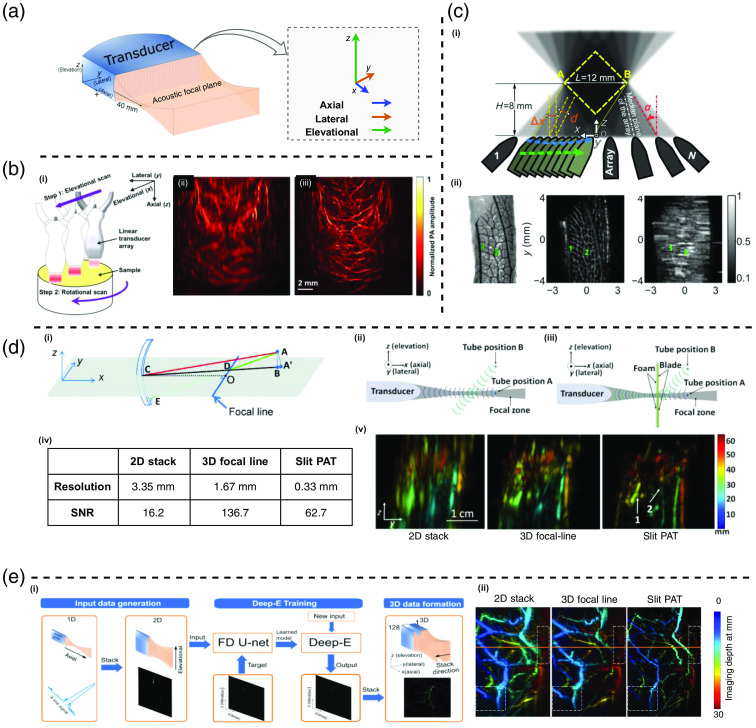
Challenges and solutions of anisotropy resolution. (a) Illustration of the poor elevational resolution due to the acoustic focus zone. Reproduced with permission from Ref. 91. (b)(i) Illustration of the system hardware setting of the IRT-PACT. The probe is fixed to a linear stage, and the object is placed on a rotation stage. (b)(ii) *In vivo* rat brain image acquired by PACT. (b)(iii) *In vivo* rat brain image acquired by IRT- PACT. Panel (b) is reproduced with permission from Ref. 58. (c)(i) Illustration of the rotate-translate scanning geometry in Ref. 93. (c)(ii) Reconstruction of a complex-shaped 3D leaf skeleton object; Image starting from the left side: ground truth image, elevational axis MAP in rotate-translate mode, and elevational axis MAP in translate-only mode. Panel (c) is reproduced with permission from Ref. 93. (d)(i) Illustration of 2D reconstruction, 3D direct reconstruction, and 3D-focal line reconstruction. A, point of reconstruction. $A^{\prime }$, the reconstructed point of A in 2D reconstruction. B, projection point of A in the x-y plane. A′C, 2D reconstruction delay. AC, 3D direct reconstruction delay. AE, 3D-focal line reconstruction delay. x-y, 2D reconstruction plane. DC equals DE. (d)(ii) Illustration of the conventional linear PACT array and its receiving aperture along elevation direction. (d)(iii) Illustration of the slit-PAT and its receiving aperture along elevation direction. Panel (d)(i) is reproduced with permission from Ref. 94. Panel (d)(ii-v) is reproduced with permission from Ref. 95. (e)(i) Illustrations of the Deep-E model data flow. (e)(ii) Illustrations of the imaging results reconstructed by conventional methods (2D stack and 3D-focal line) and Deep-E. Panel (e) is reproduced with permission from Ref. 91. FD, fully dense.

In 2D PACT, many focused transducer arrays were used for cross-sectional imaging with high temporal resolution. However, the acoustic focus of the transducer induced anisotropy resolution, an intrinsic defect of this design. Anisotropy resolution always exists even though the UTA has received perfect PA signals (e.g., well sampled, no limited view effect). The transducers in PACT are usually designed with an acoustic lens or geometrical focus to enhance their in-plane sensitivity and provide acoustic sectioning for fast 2D imaging.^59^^,^^91^^,^^92^ However, this design leads to anisotropy resolution, especially in 2D PACT imaging systems (e.g., linear array PACT).^93^^,^^94^ As shown in Fig. 5(a), the 3D resolution of a linear array can be characterized in terms of axial, lateral, and elevational resolution. The axial resolution, denoting the spatial resolution along the normal direction (x axis) of the UTA, is limited by both the speed of sound (SOS) within the acoustic medium and the bandwidth of the transducer elements. The axial resolution is the best and typically can reach half of the central acoustic wavelength. Lateral resolution, which pertains to spatial resolution along the row of transducer elements within the array (y axis), is mainly determined by the element pitch. Usually, the lateral resolution equals one acoustic wavelength, a bit worse than the axial resolution. The elevational resolution, the spatial resolution along the direction perpendicular to the axial and lateral imaging plane (z axis), is determined by the central frequency of the transducer elements and the numerical aperture (NA) of the acoustic lens or geometrical focus. The elevational resolution is usually one order of magnitude worse than the axial resolution. Anisotropy resolution also exists in PA microscopy (PAM) employing a focused transducer element. One commonly used method in PACT and PAM to achieve isotropy resolution or improve the elevational resolution is rotational scanning of the object from multiple angles to incorporate the high-frequency information from the axial or lateral direction to the elevational direction.^58^^,^^93^^,^^96^^–^^99^ In addition to the rotation, the anisotropic resolution problem can also be handled by adding a slit or using the data-driven method (deep learning).^91^^,^^95^

#### 2.2.1 Rotate-translate scanning geometry

The rotation operation mixes the poor-resolution axis (elevational axis) with the high-resolution axis (axial or lateral axis), and the translation operation ensures that there are enough overlapping files of the view area. Thus, the rotate translate-based scanning geometry can improve the elevational resolution.

PACT through inverse Radon transform (IRT-PACT) rotates the probe alone on the axial axis, mixing the elevational axis with the lateral axis. IRT-PACT introduces the Radon transform to decode the high-resolution information from the multi-direction scanned data. In IRT-PACT, as shown in Fig. 5(b)(i), the linear array probe is affixed to a linear scanning stage, and the object is placed on a rotation stage, which rotates 2 deg after each linear scanning (rotates 90 times in total). IRT-PACT employs the UBP reconstruction to generate all the B-scan frames throughout all the scanning and generates the projection along each scanning direction (elevational direction) by integrating all the tomography frames acquired within each scanning.^58^ Finally, similar to the X-ray CT, the 3D image is reconstructed through inverse Radon transform.^100^

The elevational axis projection and the inverse Radon transform make the elevational resolution almost equivalent to the in-plane lateral resolution. The results presented in Fig. 5(b)(ii–iii) depict the maximum amplitude projections (MAPs) along the depth (z) axis of the 3D images of a rat brain obtained through both conventional PACT and IRT-PACT. IRT-PACT significantly enhances elevational (vertical) resolution, producing sharper and clearer images. Further quantitative results revealed that the elevational resolution in IRT-PACT improved almost 10 times (from 1237 to 140  μm). However, in IRT-PACT, the object is scanned 90 times to obtain one 3D image, leading to a much-prolonged imaging time.

**Fig. 6:**
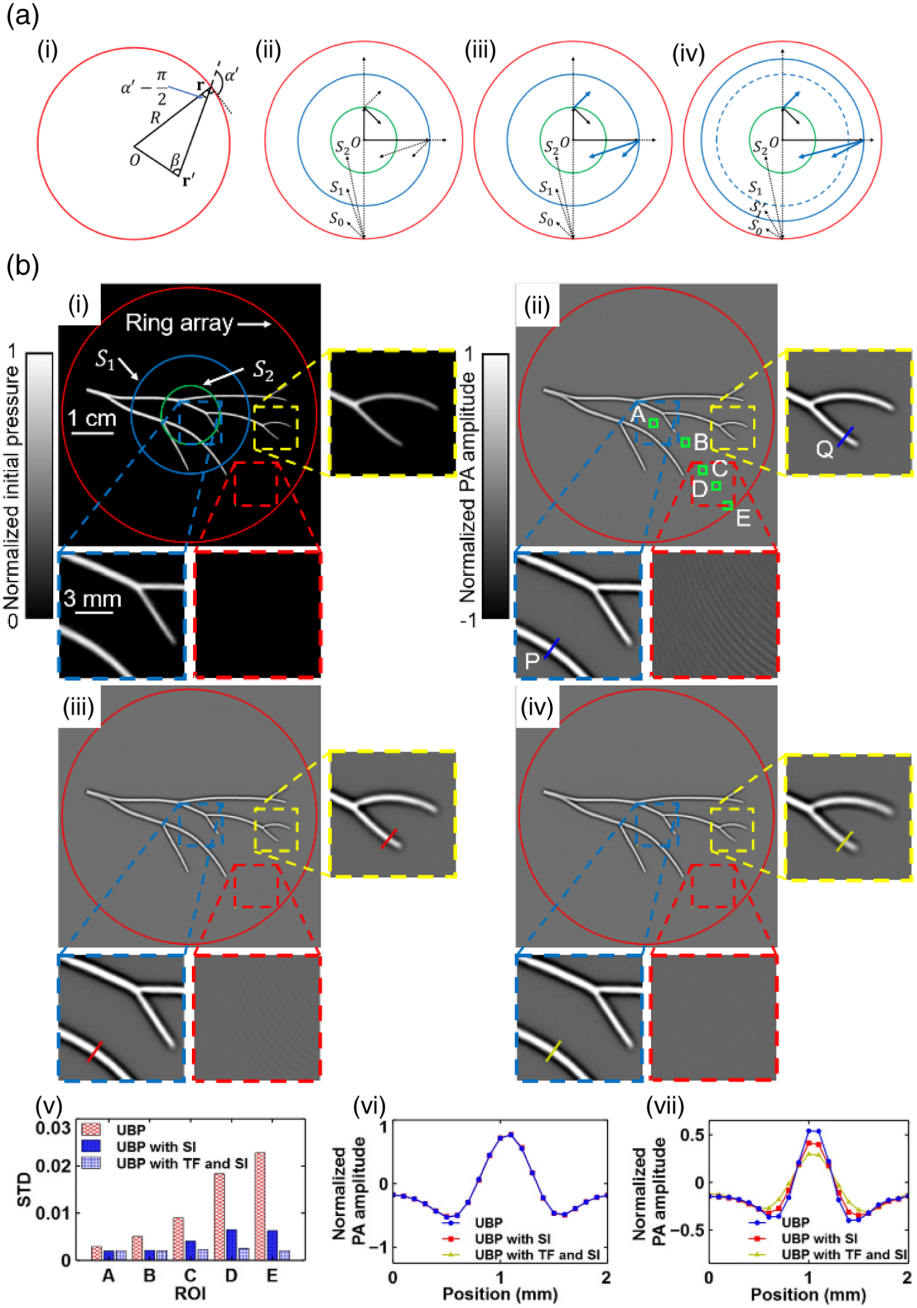
Challenges and solutions of spatial aliasing of a full ring array. (a)(i) Illustration of a full ring UTA, a transducer element r, and a source point $r^{\prime }$. (a)(ii–iv) Visualizations of three relative sizes of the three regions $S_{0}$, $S_{1}$, and $S_{2}$. The solid lines mean no aliasing, while the dotted lines mean aliasing for different location combinations of source points and reconstruction points. (a) (ii–iv) Spatial aliasing in UBP only, UBP + spatial interpolation, and UBP + spatial interpolation + temporal lowpass filtering respectively. (b)(i) Ground truth of a simple initial pressure distribution. (b)(ii) UBP reconstruction. (b)(iii) UBP with SI. (b)(iv) UBP with TF and SI. $S_{0}$, the region within the ring array. (b)(v) Comparison of the STDs in the ROIs A–E marked with the green boxes. (b)(vi–vii) Comparisons of the profiles of lines P and Q, respectively, based on the three methods. $S_{1}$, the one-way Nyquist zone. $S_{2}$, the two-way Nyquist zone. SI, spatial interpolation. TF, temporal filtering. Panels (a) and (b) are reproduced with permission from Ref. 101.

Gateau et al.^93^ rotated the probe alone on the lateral axis, mixing the elevational axis with the axial axis, as shown in Fig. 5(c)(i). The probe changes its pitch angle after each linear scanning, and the final rendered 3D image is reconstructed via 3D UBP with all the data from all the scanning data. Quotative results show that the elevational resolution can improve up to nine times. The complex 3D phantom results are shown in Fig. 6(c)(ii).

The method proposed by Gateau et al.^98^ shows good performance in improving the elevational resolution. However, generating a 3D image using all the scanning data via UBP is mathematically equivalent to reconstructing the 3D images of each scan first and then summing them up. Considering that, in the PAM field, deconvolution-based methods have been developed to solve the anisotropy resolution problem, it can be applied in PACT as well to decode the high-resolution information more efficiently to improve the performance further or reduce the number of scans.^97^^,^^98^

#### 2.2.2 3D-focal line

Xia et al.^94^ proposed 3D-focal line reconstruction to improve the elevational resolution of a focused transducer array. 3D-focal line proposed a new way to calculate the time delay, which can generate fewer artifacts and improve the elevational resolution as well as the SNR. Figure 5(d)(i) illustrates the time delays in 2D reconstruction, direct 3D reconstruction, and 3D-focal line. First, point A is projected to the focal plane (x−y plane) as point B. Second, connect point B to the center of the transducer (point C) crossing the focal line at point D. Finally, connect points A and D and extend the line to reach the transducer at point E. The line AE is used to calculate the delay time between imaging point A and the transducer. The results in Fig. 5(d)(iv) show that compared with 2D stack, 3D-focal line reconstruction improves the resolution by up to twofold.

#### 2.2.3 Slit-enabled PAT

The idea of the aforementioned 3D-focal line can be implemented in hardware by adding an additional slit to a linear PACT system at its focal line [as shown in Fig. 5(d)(ii–iii)], named slit-PAT.^95^ The slit diffracts the incoming PA waves so source points outside the transducer focal zone can still be detected, which improves the receiving aperture along the elevation direction. The thin slit is formed by two metal blades with foam covered to block the acoustic waves transmitting directly through the blade. Thus, all the PA signals received at the transducer are from the slit. The time delay in slit-PAT is the sum of the source point to the slit and the slit to the transducer, which exactly is the time delay of the 3D-focal line.

The table in Fig. 5(d)(iv) shows the elevational resolutions and the SNRs of 2D stack, 3D-focal line, and slit-PAT. A 2D stack provides the worst elevation resolution. With a 3D-focal line reconstruction, the resolution was improved by two times, and the value is close to the height of the transducer elevation focus (1.5 mm). Slit-PAT further improves resolution by almost five times to 0.33 mm, which is close to the 0.3 mm slit opening. In total, slit-PAT offers 10 times better elevation resolution than the 2D stack. Though the slit also blocks some of the incoming PA signals, the slit-PAT SNR is still four times better than that of the 2D stack. This is due to the fact that, in slit-PAT, the transducer receives the signal from all 400 scanning positions (large receiving aperture along the elevation direction). The *in vivo* experiment shown in Fig. 5(d)(v) shows that the intestine and several additional skin vessels can be identified in slit-PAT, which is hard to recognize in the 3D-focal line image.

Compared with the rotate-translate scanning methods, slit-PAT is efficient, does not need to change the scanning geometry, and can improve the elevational resolution with only a single scan. However, how to build the thin slit and make it stable may be an issue when applying slit-PAT to high-frequency probes because the slit needs to be much thinner.

#### 2.2.4 Deep learning

Deep-E is a fully dense U-Net^102^-based deep learning method designed to enhance the elevational resolution in PACT. Given that the axial and lateral resolution typically surpass elevational resolution by a significant margin, Deep-E decomposes the 3D anisotropy resolution problem into 2D (axial-elevational), specifically focusing on the axial-elevational plane during training. This approach enhances the efficiency of both simulation and model training. As shown in Fig. 5(d)(i), Deep-E takes an axial-elevation B-scan image formed by stacking all the A-lines in sequence as the input. The output of Deep-E is a 2D image with improved elevational resolution. During model inference, all generated axial-elevational images are concatenated together along the lateral direction to form the final 3D image. The pencil lead phantom shows that Deep-E can improve the elevational resolution by up to 50 times. Deep-E is also evaluated *in vivo* on humans, as shown in Fig. 5(d)(ii). Compared with conventional methods such as 2D stack and 3D-focal line, Deep-E gives shaper vascular structures with a clean background, and more importantly, Deep-E is able to extract vascular structures in deep tissue (colored in orange and red) which are difficult to recognize in the 2D stack and 3D-focal line images.^94^^,^^103^

Deep-E brings a new idea of utilizing the axial-elevational 2D training data to solve a 3D problem, which simplifies and accelerates the training data generation. Moreover, Deep-E makes the program independent from the number of elements because the experimental data were processed element by element independently in the axial-elevation plane.

### 2.3 Spatial Aliasing

Signal sampling in PACT includes both temporal and spatial sampling (SS). Temporal sampling refers to sampling a continuous-time signal to a discrete-time signal, and Nyquist sampling requires the sampling frequency to be at least twice the maximum frequency of the signal.^21^ According to different UTA geometries, the transducers around the object can be viewed as SS. Ideally, UTA should provide dense SS to satisfy the Nyquist sampling theorem,^21^^,^^27^^,^^104^ where the SS interval on the tissue surface should be less than half of the lowest detectable acoustic wavelength. If the spatial Nyquist criterion is not met, aliasing in SS causes artifacts in reconstructed images, even when the temporal Nyquist criterion has been fulfilled. Due to the high cost of a UTA with a large number of elements or limited scanning time, SS is usually spare in practice. In addition to SS, the backpropagation during the IR should satisfy the Nyquist sampling theorem as well.^101^^,^^105^ Hu et al.^101^ analyzed spatial aliasing in a ring-array-based PACT and discovered that the combination of spatial interpolation and temporal filtering can effectively mitigate artifacts caused by aliasing in either IR or SS.

#### 2.3.1 Spatial aliasing in SS

The spatial aliasing analysis of SS has the following Nyquist sampling constraints where R denotes the radius of the ring array, N denotes the total number of transducers, α denotes the angle formed by the connection of the source point and the transducer, and $\lambda_{c}$ denotes the cutoff wavelength of the cutoff frequency [Fig. 6(a)(i)].

$$\frac{2\pi R|\cos\;\alpha^{\prime }|}{N}<\frac{\lambda_{c}}{2}.$$

After transforming this inequality to a constraint for the source point location $r^{\prime }$ via the Law of Sines, we get the smallest upper limit of $r^{\prime }$

$$r^{\prime }<\frac{N\lambda_{c}}{4\pi }.$$

The region within this constraint is defined as the one-way Nyquist zone $S_{1}$. For any source points inside $S_{1}$, there is no spatial aliasing during SS because the sampling spacing is less than half of the lower cutoff wavelength [Fig. 6(a)(ii)].

#### 2.3.2 Spatial aliasing in IR

Similar to the spatial aliasing analysis of SS, IR also has the Nyquist sampling constraints, and the final result can be written as

$$r^{\prime \prime }+r^{\prime }<\frac{N\lambda_{c}}{4\pi },$$

where $r^{\prime }$ is the source point and $r^{\prime \prime }$ is the reconstruction point.

A region $S_{2}$ within the following constraint is defined as the two-way Nyquist zone.

$$S_{2}=\{r^{\prime }||r^{\prime }|<\frac{N\lambda_{c}}{8\pi }\}.$$

Spatial aliasing in IR depends on the locations of the source point and the reconstruction points. Spatial aliasing does not appear for objects and reconstruction locations inside $S_{2}$ but appears for other combinations of objects and reconstruction locations [Fig. 6(a)(ii)].

#### 2.3.3 Spatial antialiasing in SS and IR

Spatial aliasing solely in IR but not in SS can be well addressed by spatial interpolation. To extend the region $S_{2}$, we can numerically double the number of detection elements $N^{\prime }=2N$ based on the interpolation. Thus, the new two-way Nyquist zone ${S^{\prime }}_{2}$ becomes the same as $S_{1}$, indicating that spatial interpolation successfully removes spatial aliasing in IR [Fig. 6(a)(iii)]. Hakakzadeh et al.^106^ stated that reducing the number of transducers causes artifacts, but the structure similarity improved by 30% after interpolation. Wang et al.^107^ tested different interpolation methods and proposed an interpolation method named extremum-guided interpolation, which does not require complex calculations and can effectively improve the quality of PA reconstruction under sparse sampling. However, interpolation cannot recover the information lost for the spatial aliasing outside the $S_{1}$ because SS has aliasing.

Hu et al.^101^ introduced temporal lowpass filtering to eliminate the spatial aliasing in SS, given that $S_{1}$ is defined by the cutoff wavelength $\lambda_{c}$ and a temporal lowpass filter replaces $\lambda_{c}$ with a longer wavelength ${\lambda^{\prime }}_{c}$. Thus, the one-way Nyquist zone is extended [Fig. 6(a)(iv)] through temporal lowpass filtering at the expense of spatial resolution, blurring the reconstructed images. To balance between spatial antialiasing and high resolution, Hu et al.^101^ proposed radius-dependent temporal filtering: for the region within $S_{1}$, the PA raw signal should be interpolated and perform reconstruction; for the region outside $S_{1}$, a temporal lowpass filter should be applied to the raw signal and then perform spatial interpolation and reconstruction.

The spatial interpolation and radius-dependent temporal filtering are evaluated in Fig. 6(b). The reconstruction quality is improved by spatial interpolation, and the aliasing artifacts are further mitigated by temporal filtering.

It should be noted that even though there is no limited view or anisotropy resolution issue in the PACT system, due to the insufficient SS, the best reconstruction quality can be guaranteed only within the two-way Nyquist zone $S_{2}$. After spatial interpolation, the well-reconstructed area can be enlarged to the one-way Nyquist zone $S_{1}$. The concepts of one-way and two-way zones are useful to guide people in system design. For example, based on the $S_{1}$, a 10 MHz full-ring array should have at least 1024 elements to have a perfect reconstruction area of 24 mm in diameter, which is enough for whole mouse imaging.

## 3 Acoustic Heterogeneity (SOS Mismatch)

**Fig. 7:**
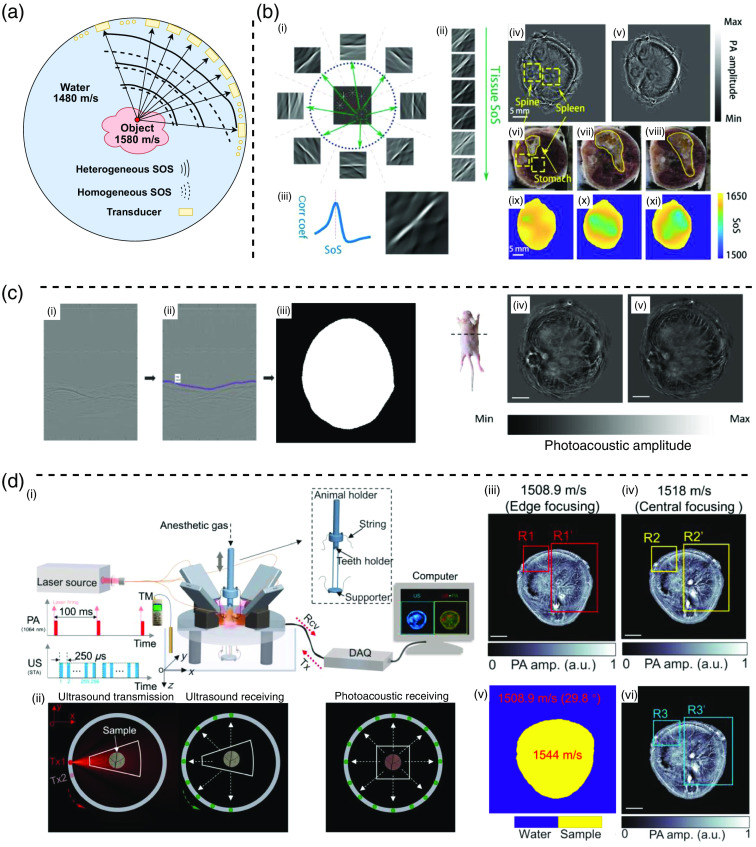
Challenges and solutions of acoustic heterogeneity. (a) Heterogeneous SOS affects the time delay of the PA signal. (b)(i) MSFC divides the ring array into eight subgroups and reconstructs a region with different SOS independently. (b)(ii) Illustrations of reconstructions with different SOS. MSFC measures the correlation coefficients to evaluate SOS marching. (b)(iii) SOS matching results. The peak is assumed to be the mean SOS along the direction through the two opposite subgroups. (b)(iv) *In vivo* animal imaging result reconstructed by MSFC. (b)(v) *In vivo* animal imaging result reconstructed with single (homogeneous) SOS. (b)(vi–viii) The cryotomy photos of the mouse’s stomach. Spine and spleen are marked by yellow dashed line boxes, the corresponding region in the cryotomy photo. (b)(ix–xi) The estimated SOS distribution generated MSFC roughly at the three cryotomy layers shown in panel (b)(vi–viii). Panel (b) is reproduced with permission from Ref. 113. (c)(i) Visualization of the raw transducer data. (c)(ii) The identified object surface signal. (c)(iii) The reconstructed object shape based on the identified object surface signal in panel (c)(ii). (c)(iv) *In vivo* animal imaging result reconstructed with single SOS. (c)(v) *In vivo* animal imaging result reconstructed by the dual SOS reconstruction. The scale bar is 5 mm. Panel (c) is reproduced with permission from Ref. 114. (d)(i) Illustrations of the system hardware setup of the ADS-USPACT. (d)(ii) Illustrations of the US transmission and the US/PA data acquisition. The red dot represents the sequentially activated transmission element, and the green dots represent the receivers. (d)(iii–iv) Reconstruction results with different SOS. The single SOS reconstruction cannot achieve global focus. (d)(v) The estimated dual SOS map. (d)(vi) The dual SOS reconstruction image generated by ADS-USPACT. The scale bar is 4 mm. Panel (d) is reproduced with permission from Ref. 115.

In PACT reconstruction, a crucial factor is the distribution of acoustic properties (e.g., SOS and acoustic impedance) within the acoustic propagation pathway.^108^^–^^112^ SOS plays an important role as it directly determines the time arrivals of PA signals [Fig. 7(a)]. In this review paper, we mainly focus on the SOS mismatch although acoustic heterogeneity could be broad. Notably, the SOS distribution along the acoustic propagation path is inherently heterogeneous, especially for *in vivo* imaging, exhibiting variations among the coupling medium, water (∼1480  m/s at 20°C), and tissue (∼1580  m/s). Any misalignment in the SOS setting can lead to inaccuracies in the reconstructed initial pressure, causing artifacts in the reconstructed images.^116^

The SOS mismatch issue is particularly pronounced in the full-ring array-based PACT compared with the linear array systems.^27^ Due to the symmetry of a full-ring geometry, the reconstruction is contributed by the transducers located at two opposite sides of the ring. Consequently, the SOS setting should be very precise; otherwise, the source points reconstructed from transducers on opposite sides may fail to align properly, causing artifacts such as shadows, arcs, and double copies. As for the linear array-based PACT systems, though they also could suffer from the SOS mismatch issue, the reconstruction artifacts are not as severe as the artifacts from full-ring array-based PACT.

### 3.1 Single SOS Searching

Reconstructing PACT images while assuming a single, universal SOS simplifies the process despite the fact that this assumption is not entirely correct and leads to reconstruction artifacts. Thus, researchers often opt for this simplification and try to find the optimal SOS value that has the least artifacts.^117^

### 3.2 Joint Reconstruction

Joint reconstruction (JR) is an iterative model-based method that reconstructs the initial pressure and the SOS distribution simultaneously.^110^^,^^118^ The two subproblems are solved alternatively until a convergence condition is satisfied. The reconstruction of the initial pressure is a convex optimization problem since the objective function is convex for fixed SOS. However, the SOS distribution reconstruction is a non-convex problem. Huang et al.^119^ found that accurate JR images were not produced when the spatially variant absorbed optical energy density distribution (initial pressure) is deficient, but the jointly reconstructed initial pressure could be more accurate than the one reconstructed with a constant SOS. In addition, the jointly reconstructed initial pressure was more accurate than the jointly reconstructed SOS distribution, which indicated that the inverse problem of reconstructing SOS distribution is more unstable compared with the reconstruction of initial pressure.^119^

Another JR solver is adaptive PACT.^120^ Cui et al.,^120^ inspired by adaptive optics, tried to introduce the indirect wavefront measurement idea to PACT to solve the JR problem. The image is reconstructed patch by patch. Within each patch, the wavefront distortion is almost identical (“isoplanatic patch”) and can be extracted from the local point spread function (PSF). Similar to the “phase diversity,” the local PSF, which has long been regarded as an unknown, can be computationally found from a stack of local images reconstructed with different delays.^121^ Thereby, the full image can be better focused via piecewise deconvolution. After the wavefronts of all the patches are determined, they can be used collectively to compute the global SOS map. Thus, it bypasses the cumbersome global searching of the SOS map and improves the stability and reliability of the solution.

### 3.3 Multi-segmented Feature Coupling

As shown in Fig. 7(b)(i), it was demonstrated that SOS mismatch leads to a misalignment of the reconstructed source points from opposite transducers. Thus, the reconstruction results of opposite transducers can serve as a good indicator to evaluate the accuracy of the SOS setting. The feature coupling method divides the transducers into two semicircles and reconstructs two images independently.^122^ The SOS distribution is iteratively adjusted to maximize the correlation between the two reconstructed images. Building upon the concept of feature coupling, multi-segmented feature coupling (MSFC) divides the ring array into eight subgroups. Two subgroups located at opposite sides reconstruct a region with different SOS.^113^ MSFC measures the correlation coefficients between the two reconstructed images from two opposite subgroup transducers [Fig. 7(b)(ii)]. The peak determines the mean SOS along the direction through the two opposite subgroups [Fig. 7(b)(iii)].

The results are shown in Fig. 7(b)(vi–viii). If reconstructed properly, a vessel perpendicular to the imaging plane will be reconstructed as a point [Fig. 7(b)(iv)]. If the SOS estimation is wrong, the vessel will be distorted into a ring shape [Fig. 7(b)(v)]. The estimated SOS distributions [Fig. 7(b)(ix–xi)] show that the SOS of the stomach region (coconut oil) is significantly lower, with the profile and location roughly matching those in the cryotomy photos.

MSFC optimizes the SOS distribution based on the feature coupling, which avoids cumbersome matrix calculations and saves a lot of computation time (compared with JR). However, the feature coupling relies on the object features, which may limit its generalizability, as not all tissue areas are rich in features suitable for SOS estimation. In addition, the operator is asked to select the features and draw boundaries manually. A fully automatic method would be much preferred for future practical applications.

### 3.4 Dual SOS Reconstruction

To simplify the SOS map estimation and reconstruction while improving the image quality, the dual SOS assumption has been adopted in PACT reconstruction.^27^ In dual SOS reconstruction, a binary SOS map is created, consisting of two SOS values representing the water area and the tissue object area. This simplification is made based on the premise that the SOS difference within soft tissue is relatively small compared with the difference between water and tissue. The effectiveness of dual SOS reconstruction hinges on two key components: (1) estimated object boundary and (2) estimated SOS values.

#### 3.4.1 Object surface PA signal detection

Reference 114 utilized a U-Net^123^ model to identify the object PA surface signal in raw data and reconstruct the object shape [shown in Fig. 7(c)(i–iii)]. However, in this method, the two SOS values assigned to the binary SOS map are predefined as 1480 and 1570  m/s, which are two commonly used preset SOS values in water and soft tissues.^124^ The results shown in Fig. 7(c)(iv–v) demonstrate the benefits of the dual SOS approach. It not only corrects the SOS distribution but also suppresses the artifacts. The idea of utilizing the object surface PA signal to reconstruct the object boundary is promising because the surface signal only travels in water, and the SOS in water is known. However, the SOS in the object is also preset by the operator, which may not be the best solution. The idea of utilizing the surface PA signal can be further developed to adaptively estimate the SOS in the object.

#### 3.4.2 US + PACT

Instead of estimating the SOS distribution, the object boundary and the optimal SOS can also be detected by US imaging. Jose et al.^125^ proposed passive element-enriched PACT where a passive point source was introduced to profile the SOS distribution. References 115 and 126 integrated active US source and PA imaging to develop an adaptive dual-speed US and PACT (ADS-USPACT) system that automatically segments the object boundary and determines the optimized SOS values. In ADS-USPACT, the SOS in water is determined by the water temperature, and the object boundary is detected by US imaging. To find the optimal SOS in the object, ADS-USPACT searches for the maximum coherence factor among the US signals at various sample SOS values.

Figure 7(f)(iii–vi) provides a visual comparison between ADS-USPACT and single SOS reconstruction. Single SOS cannot achieve global improvement in imaging quality, e.g., 1508.9  m/s makes boundary vessels in focus, and 1518  m/s makes the central vascular features in focus, but there is no optimal single SOS that can make the whole object focus. ADS-USPACT, on the other hand, can keep both the boundary and the central vessels in focus.

ADS-USPACT performs good dual SOS reconstruction quality at the expense of additional US imaging hardware and reconstruction overhead. Dual SOS reconstruction is a potential solution as it simplifies the SOS distribution and can generate high-quality images. However, it still needs to be further developed to make it computational- and hardware-friendly.

### 3.5 Deep Learning

**Fig. 8:**
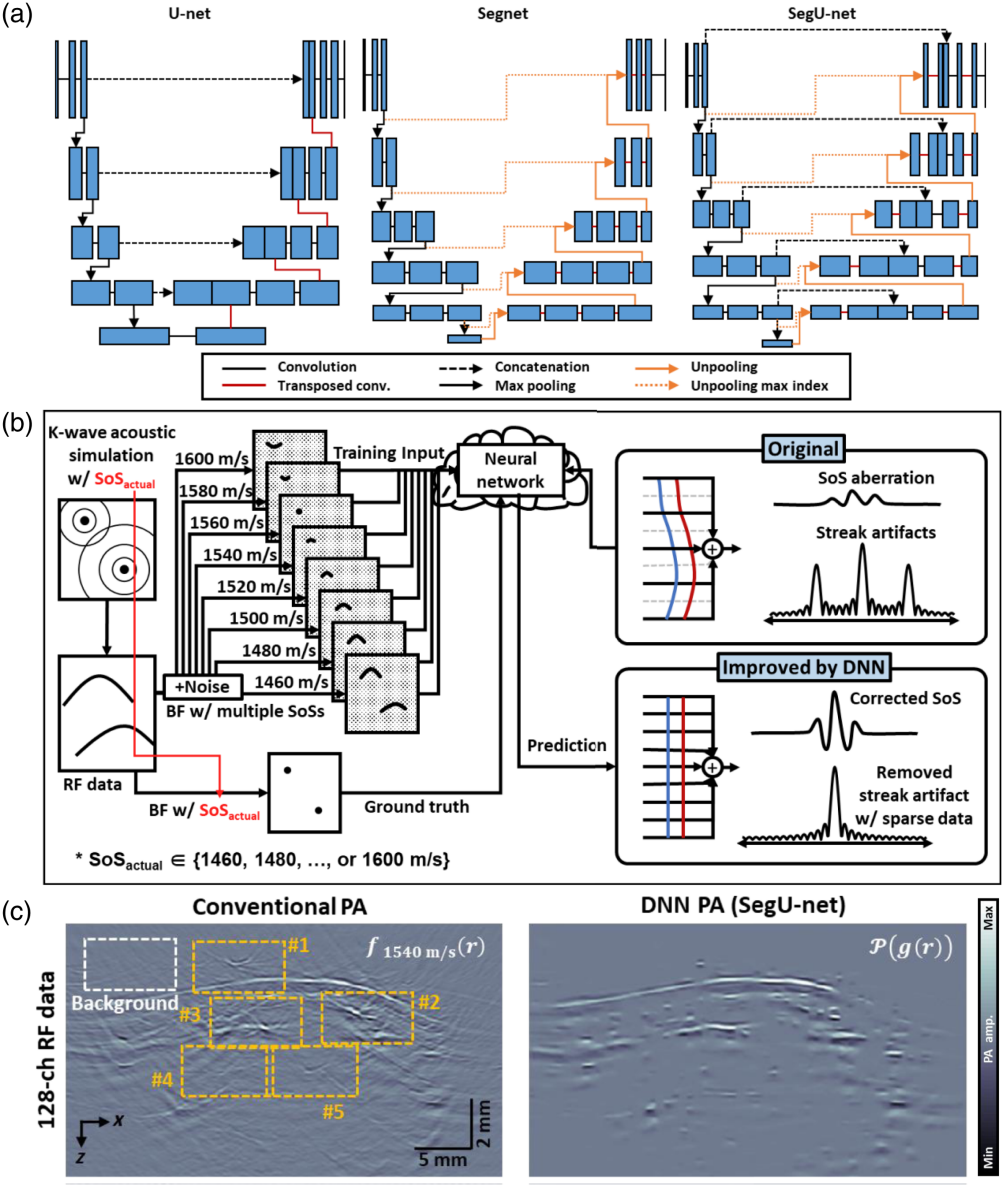
(a) Model architecture of U-net, Segnet, and SegU-net. (b) Illustration of the data flow of SegU-net. The model takes reconstruction with different SOS as input. Based on the training dataset, the deep neural network is trained to correct the SOS aberration and streak artifacts in the PA images. (c) *In vivo* human forearm PA images reconstructed via conventional beamforming (left) and the SegU-net (right). BF, beam forming. All panels are reproduced with permission from Ref. 117.

Though linear array PACT systems are not as sensitive to the SOS mismatch as ring array PACT systems, SOS mismatch also causes artifacts in linear array PACT images, e.g., a point source may be reconstructed as an arc if the SOS is not matched [Fig. 8(c)]. Reference 117 proposed a deep learning-based SOS calibration method. They evaluated their method on U-Net, Segnet, and a proposed hybrid model of U-net and Segnet, named SegU-net [Fig. 8(a)]. As shown in Fig. 8(b), the input data are a group of reconstructed images based on eight different single SOS reconstructions, starting from 1460 to 1600 m/s, and the target is the corresponding ground truth image. Though all the training data are generated in a homogeneous medium by K-wave simulation, SegU-net shows its ability to reconstruct and alleviate artifacts in a heterogeneous medium. Figure 8(c) shows the *in vivo* human forearm PA imaging results reconstructed by the single SOS reconstruction and the SegU-net. The SoS aberration and streak artifacts are remarkably reduced in the SegU-net-corrected PA images. We expected that deep learning can be further extended to ring arrays or 3D geometry arrays such as planar or spherical arrays.

## 4 Fluence Correction

**Fig. 9:**
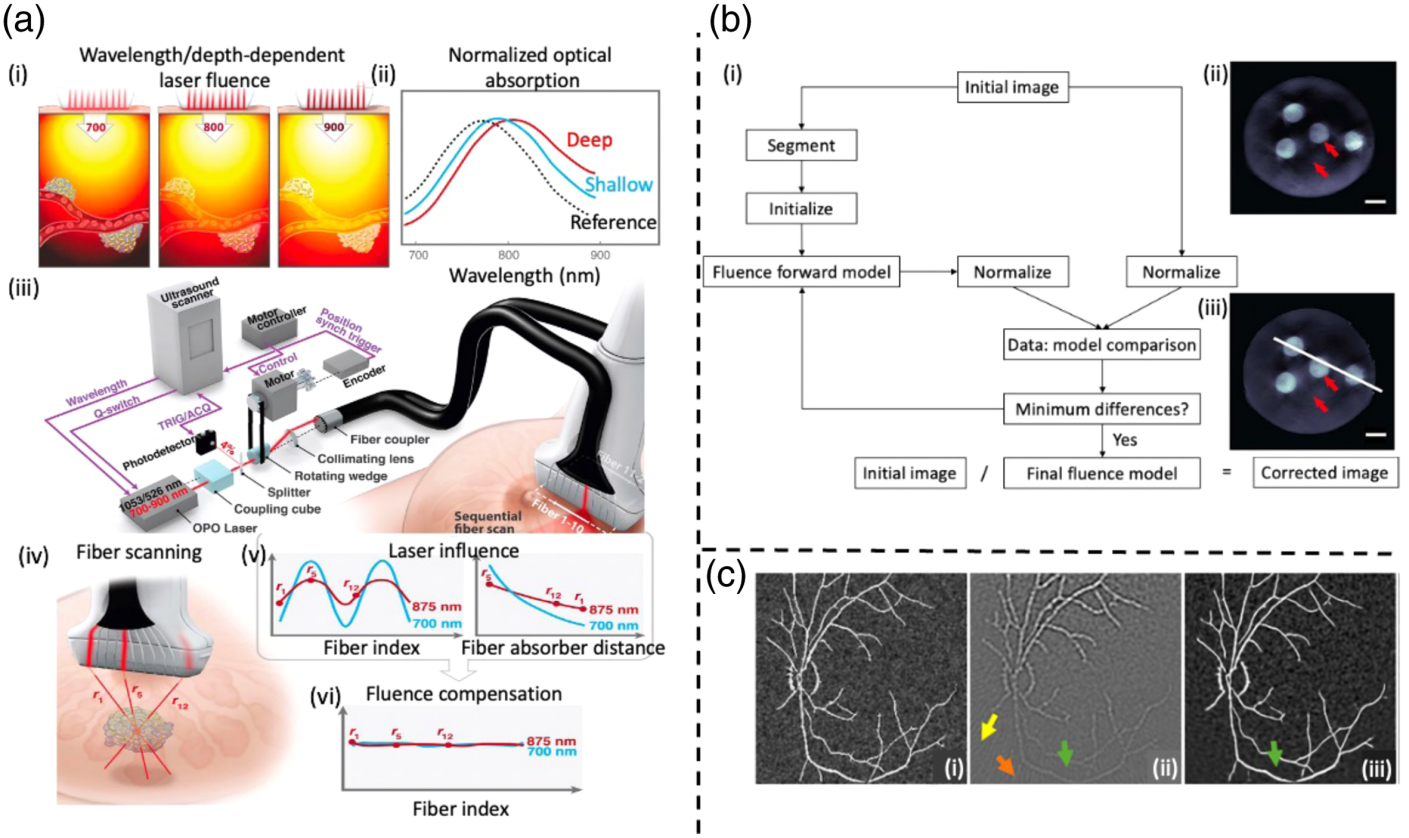
Fluence incorrection challenges and solutions. Reprinted with permission from Refs. 127–129. (a)(i) Wavelength- and depth-dependent optical fluence in tissue can significantly influence optical absorption spectrum measurements. (a)(ii) Spectrum of gold nanorods shifts as image depth increases. (a)(iii) Scanning system comprises a kHz-rate, wavelength-tunable diode-pumped laser, a fiber delivery system, and a US scanner, with the laser emitting variable-wavelength pulses triggered by the scanner while maintaining a high repetition rate. (a)(iv) Light from various fibers travels varying distances to reach a target. (a)(v) The amplitude of partial PA images, obtained by single-fiber irradiation, is influenced by light absorption and scattering in tissue, and it is dependent on the distance between each fiber and a typical absorber within the imaging field. (a)(vi) Real-time compensation to get wavelength-independent fluence. (b)(i) Optimization process for extracting the light fluence distribution and conducting fluence correction. (b)(ii) Initial image. Red arrows highlight a decrease in image intensity caused by optical attenuation (b)(iii) Fluence-corrected image. The scale bar is 3 mm. (c)(i) Reference image. (c)(ii) TR reconstruction image. (c)(iii) Fluence correction result using the U-Net deep learning model.

The amplitude of the PA signal depends on both the optical absorption and laser fluence. However, it is important to note that tissue attenuation varies with wavelength, as illustrated in Fig. 9(a)(i). Consequently, when estimating the optical absorption from a PA image, accuracy can be compromised, resulting in significant changes in shape and a shift in the wavelength of maximum absorption, particularly in deeper tissue regions, as shown in Fig. 9(a)(ii). Optical attenuation in fluence measurements can distort PA signals, potentially impacting the accuracy and the ability to quantitatively interpret the resulting images.^130^ Implementing fluence correction techniques becomes essential to mitigate these challenges and ensure precise quantification. In this section, spectroscopic PA imaging, iterative optimization methodology, and deep learning models have been employed to achieve fluence compensation.

### 4.1 Spectroscopic PA Imaging

Spectroscopic imaging approaches are proposed for the automated correction of wavelength-dependent fluence variations.^127^^,^^131^^,^^132^ Kim et al.^131^^,^^132^ succeeded in correcting the wavelength-dependent fluence distribution and demonstrated its performance in phantom studies using a conventional handheld US probe and validated the performance based on phantom studies. Jeng et al.^127^ proposed that 10 fibers are evenly distributed along each elevational edge of the US transducer array, as depicted in Fig. 9(a)(iii). Unlike previous systems that simultaneously delivered laser pulses into all fibers in a bundle, this setup sequentially couples light into individual fibers. Partial PA IR is generated for each laser pulse, contributing to the estimation of laser fluence. Importantly, as shown in Fig. 9(a)(iv), light emerging from different fibers travels distinct distances to reach a target. Figure 9(a)(v) illustrates how the PA signal amplitude varies with fiber index, while the upper right plot in Fig. 9(a)(v) showcases the PA signal loss with distance due to light attenuation, resulting in computational error. It is worth noting that fluence losses with depth will differ across different wavelengths. Amplitude variations concerning the distance between any pixel and the source for numerous points are acquired, where these points exhibit partial PA image amplitudes above the noise floor. These measurements serve as input data for the fluence reconstruction process, which leverages the light diffusion model. With this procedure repeated for all wavelengths, fluence can be disentangled from the PA image, leading to the retrieval of the true light absorption spectrum of molecular absorbers, as shown in Fig. 9(a)(vi). This method has demonstrated its superiority in phantoms, *ex vivo* and *in vivo* experiments.^127^

### 4.2 Iterative Optimization

Iterative optimization methodology can be applied for fluence correction.^133^^–^^135^ The optimization process [Fig. 9(b)(i)] begins with a 2D reconstruction of an initial image [Fig. 9(b)(ii)] using model-based acoustic reconstruction, where the low-image intensity caused by optical attenuation is along the red arrows, as shown in Fig. 9(b)(ii). To expedite the optimization, the initial image is segmented into regions based on prior knowledge of the object structure, with constant optical properties (including absorption and scattering coefficients) that can be tuned during optimization within each region. This optimization problem utilizes a δ-Eddington approximation of the radiative transfer equation as the light fluence model. Notably, artifacts resulting from optical attenuation in Fig. 9(b)(ii) are effectively eliminated after fluence correction, as shown in Fig. 9(b)(iii). In this research, invariant system response and parameters are assumed at first to carry out phantom experiments, but it is hard to use the same parameter settings in future *in vivo* experiments.^128^ Another work proposed by Naser et al.^134^ is to combine finite-element-based local fluence correction with SNR regularization and validate its performance in both *ex vivo* and *in vivo* experiments.

### 4.3 Deep Learning

A deep learning approach can be used to recover the optical absorption maps by correcting for the fluence effect.^90^^,^^136^^–^^138^ Figure 9(c)(i) presents the reference ground truth image, while in Fig. 9(c)(ii), the image is reconstructed using TR, which is blurred and noisy. In Fig. 9(c)(ii), the yellow arrow points to noticeable reconstruction artifacts, and the orange arrow highlights the impact of fluence on small vasculature in deep tissue regions. In addition, there is the presence of undesirable vasculature, indicated by a green arrow, in the reconstructed images. The impact of optical fluence on PA images can be removed by employing end-to-end map training as a supervised learning problem. A neural network is trained to minimize the loss function to obtain the fluence-corrected images. Figure 9(c)(iii) displays the corresponding reconstruction outcomes using the U-Net DL model, where the shape of vasculature is successfully recovered in deep regions.^129^ DL models proposed by Arumugaraj et al.^138^ were shown to be ∼17 times faster than solving the diffusion equation for fluence correction. Complex, and non-homogeneous medium, background tissue properties are all considered for fluence compensation, which is critical for future clinical usage.^129^^,^^138^ Chen et al.^137^ proposed a DL approach to recover the optical absorption coefficients of biological tissues and verified it in phantom experiments, while Arumugaraj et al. validated their DL models with both *in silico* and *in vivo* datasets.

## 5 Conclusion

In summary, although 2D PACT has been widely used in pre-clinical studies and clinical translations,^139^^–^^141^ it still faces challenges for quantitative measurements. These challenges encompass issues such as limited view,^79^^,^^142^ anisotropy in resolution along varying spatial axes,^126^^,^^143^ spatial aliasing,^101^ reconstruction artifacts caused by acoustic heterogeneity,^144^^,^^145^ and quantitative spectral unmixing with fluence correction.^146^^,^^147^ Effectively mitigating these challenges necessitates innovative strategies spanning the domains of hardware engineering,^20^^,^^148^ signal processing methodologies,^115^^,^^149^ and deep learning paradigms.^78^^,^^85^^,^^144^

The challenge of limited view imaging, stemming from the inherent constraints of linear/planar transducer arrays, has been addressed through diverse methodologies. These solutions have included the deployment of acoustic reflectors and ultrasonic heating encoding, iterative optimization, and the integration of advanced deep-learning approaches. These interventions are purposefully devised to expand the scope of IR, even when confronted with linear detectors possessing limited viewing angles, thus facilitating a marked enhancement in imaging fidelity. DL provides a solution to address the limited view problem, allowing for precise high-resolution PACT reconstruction even with sparse viewing angles. Enhancing DL methods, such as incorporating transformers, offers a means to handle long-range dependencies effectively.

Anisotropy resolution issues have been methodically approached through techniques that include rotate-translate scanning, slit-PAT, and deep learning methodologies. These tactical measures substantially augment elevational resolution and achieve isotropic resolutions in the resultant images. This enables a more lucid visualization of intricate structures inherent in biological tissues. However, those methods still need to be further developed. The rotate-translate scanning methods require multi-angle scanning, which is too time-consuming. Slit-PAT may have some issues when applying to the high-frequency probe as the slit needs to be much thinner.

Spatial aliasing issues can be mitigated by spatial interpolation and temporal lowpass filtering. However, there is still a trade-off between spatial antialiasing and high-resolution reconstruction for regions outside the one-way Nyquist zone, which could be addressed via location-dependent antialiasing but with significantly increased computational cost.^105^

Acoustic heterogeneity (SOS mismatch), characterized by disparities in the SOS within distinct tissue types, has been systematically addressed via innovative techniques, such as JR, dual SOS reconstruction, and deep learning-driven SOS calibration. These strategies rectify artifacts associated with SOS variations and refine image quality, thereby facilitating a more precise and reliable interpretation of PA images. Though current solutions have proved that the reconstructed image qualities can be improved a lot at the expense of additional hardware or huge computation overheads (iterative methods), an efficient and adaptive solution is necessary to address the SOS mismatch problem in future research.

Incorporating fluence correction has emerged as an imperative facet of PACT to account for fluctuations in laser fluence and its interaction with tissue absorption properties. Sequential fiber-based data acquisition, iterative optimization methodologies, and deep learning models have been adeptly employed to disentangle fluence-induced effects from PA images. These endeavors culminate in more accurate representations of absorption characteristics and bolster the credibility of quantitative analyses. However, implementing real-time fluence correction is still challenging but crucial for dynamic imaging scenarios. Methods that can adapt to changes in tissue geometry and optical properties in real time are desirable. Another concern is that fluence correction may need to be adapted for different wavelengths used in multispectral PACT, where each wavelength experiences distinct absorption and scattering properties in tissues.

In summative contemplation, the realm of PA imaging is continually evolving,^150^^–^^153^ with advancements spanning both hardware and software domains that are meticulously tailored to surmount the intrinsic impediments. These strides hold the promise of significantly enhancing the accuracy, resolution, and reliability of PACT, positioning it as an invaluable tool in diverse biomedical applications, particularly for the high-fidelity imaging of biological tissues and structures.

## Data Availability

Data sharing is not applicable to this article as no new data were created or analyzed.
